# Efficacy of physiotherapeutic interventions in the management of functional constipation in pediatric and adolescent populations: a systematic review and meta-analysis

**DOI:** 10.1007/s00431-026-06831-8

**Published:** 2026-04-10

**Authors:** Esther M. Medrano-Sánchez, Marta Díaz-Gutiérrez, Paula García-Polo, Esther Diaz-Mohedo

**Affiliations:** 1 Departamento de Fisioterapia, Universidad de Sevilla, Calle Avenzoar, 9, 41009 Sevilla, Spain; 2 Departamento de Fisioterapia, Universidad de Málaga, Avda. de Cervantes, 2, 29016 Málaga, Spain; 3 Physiotherapist, Medical Center Rubén García, Sevilla, Spain; 4 Physiotherapist, Zaidafisiopelvic, Sevilla, Spain

**Keywords:** Functional constipation, Physiotherapy, Pelvic floor, Pediatrics

## Abstract

To evaluate the efficacy of physiotherapeutic interventions in the management of functional constipation (FC) in pediatric and adolescent populations, and to analyze the methodological quality of the available evidence. A systematic review of randomized controlled trials (RCTs) published in PubMed, Cochrane Library, Embase, and Web of Science was conducted (February–March 2025). Studies including participants aged 18 years diagnosed with FC, treated with pelvic floor physiotherapy versus conventional medical treatment, were included. Methodological quality was assessed using the PEDro scale (Physiotherapy Evidence Database). Seven clinical trials were included in the meta-analysis with sample sizes ranging from 45 to 400 participants (total *n* = 775). The interventions covered pelvic floor exercises, biofeedback, telerehabilitation, visceral mobilization, and electrostimulation. The majority showed improvements in painful defecation, fecal consistency, and quality of life, with less consistent results in defecatory frequency (MD = 1.00; 95% CI: 0.35 to 1.65). However, the marked heterogeneity in protocols (type, intensity, duration), variability in assessment instruments, and frequent co-intervention with polyethylene glycol (PEG) hinder comparison between studies and limit external validity. Follow-ups shorter than 12 months and the absence of blinding of participants and therapists constitute additional risks of bias.

*Conclusions*: Pelvic floor physiotherapy appears to be a promising adjuvant intervention in the treatment of pediatric FC, with positive effects on symptoms and quality of life. Nevertheless, multicenter trials with standardized protocols, large samples, and prolonged follow-up are required to confirm its efficacy and long-term sustainability.

**Table:** 

**What is Known:** •*Despite the clinical use of physiotherapy, there was no quantitative synthesis integrating its efficacy against standard medical treatment, nor had its specific effect size on defecatory pain and quality of life been established in the pediatric population.*
**What is New:** •*This meta-analysis confi rms that pelvic fl oor physiotherapy is an eff ective adjuvant with a large effect size, demonstrating consistent improvements in reducing painful defecation and enhancing the biopsychosocial dimensions of quality of life.*

**Supplementary Information:**

The online version contains supplementary material available at 10.1007/s00431-026-06831-8.

## Introduction

Functional constipation (FC) is one of the most frequent gastrointestinal disorders in childhood and adolescence, with an estimated prevalence between 0.7% and 29% depending on the geographical region [1–3]. It is characterized by a decrease in the usual frequency of bowel movements, often accompanied by difficulty evacuating, the presence of hardened stools, painful defecation, and/or a persistent feeling of incomplete evacuation [1, 2]. Consequently, the quality of life is compromised, even affecting social relationships, which underscores the need to establish a safe and effective treatment for this population.

The initial management in cases of FC focuses on educating the patient and their environment, demystifying misconceptions about the clinical presentation, promoting lifestyle modifications, and establishing appropriate defecatory habits. Polyethylene glycol (PEG) is the treatment of choice for disimpaction and maintaining intestinal transit, especially in children with neurological and behavioral maturity equivalent to or greater than 4 years [1]. Regarding side effects, the use of PEG in very high doses may be associated with an increase in fecal incontinence (FI) [3–6]. In addition to pharmacological treatment, non-pharmacological therapies such as cognitive-behavioral therapy, dietary changes, probiotics, and pelvic floor physiotherapy are being studied, highlighting biofeedback training [7]. Available studies suggest that interferential electrical stimulation, applied to the abdominal or pelvic floor region and combined with exercises, could constitute an effective and safe intervention, with significant improvements in general FC symptoms [8, 9]. Similarly, abdominal manual therapy has been identified as a promising alternative, showing an improvement in stool frequency and consistency, as well as quality of life, compared to placebo in controlled trials [10]. Both lifestyle-associated components and dysfunctions in rectal motility and pelvic floor coordination appear to play a fundamental role in the pathophysiology of the disorder [1]. Therefore, pelvic floor physiotherapy is gaining increasing importance.

Given that FC is considered a highly prevalent gastrointestinal disorder, and that the therapeutic approach continues to be based primarily on medications—despite the possible associated adverse reactions—the objective of the present systematic review is to analyze the available evidence regarding the efficacy of pelvic floor physiotherapy in the management of FC. In particular, the aim is to determine its impact on symptom control and improvement in quality of life in the pediatric and adolescent population.

## Materials and methods

### Study design

A systematic review of clinical trials was conducted in accordance with the guidelines established in the PRISMA declaration (Preferred Reporting Items for Systematic Reviews and Meta-Analyses). This review was registered in the International Prospective Register of Systematic Reviews (PROSPERO) under identifier CRD420251105672.

The research question formulated was: “Does physiotherapy constitute an effective therapeutic option for improving symptom control and quality of life compared to conventional treatment in children and adolescents with FC?”.

### Data sources

An exhaustive bibliographic search was carried out between February and March 2025 in the PubMed, Cochrane Library, Embase, and Web of Science databases. Health Sciences Descriptors (DeCS) and Medical Subject Headings (MeSH) related to constipation, children, pelvic floor, biofeedback, exercise, physiotherapy, conservative treatment, and quality of life were used, combined using the Boolean operators “AND” and “OR”.

Examples of the search strategies used included: PubMed/Cochrane: (“Functional Constipation” AND Children AND (Biofeedback OR Exercise OR Physiotherapy OR “Quality of Life” OR “Interferential Electrical Stimulation” OR “Conservative Treatment”)); Embase: (‘functional constipation’ AND children AND (‘biofeedback’/exp OR ‘exercise’/exp OR ‘physiotherapy’/exp OR ‘quality of life’/exp OR ‘interferential electrical stimulation’ OR ‘conservative treatment’/exp)); Web of Science: (“Constipation” OR “Functional Constipation”) AND (“Physical Therapy Modalities” OR “Pelvic Floor Muscle Training” OR “Biofeedback” OR “Exercise Therapy” OR “Pelvic Floor Rehabilitation” OR “Electrostimulation”) AND (“Pelvic Floor” OR “Defecation Disorders”) AND (“Pediatrics” OR “Children” OR “Adolescents”).

(Annex 1. Search strategies used for the different databases. Available as Supplementary Material).

### Study selection

All studies included in this review had to be randomized controlled trials (RCTs), with participants aged 18 years or less, clinically diagnosed with FC, and treated using any modality of pelvic floor physiotherapy in the experimental group (EG). RCTs whose participants had other associated pathologies (digestive, neurological, metabolic, or others) were excluded.

The main outcome was the presence or absence of FC. Other outcomes assessed were defecation frequency, painful defecation, stool consistency, and quality of life.

### Data extraction and methodological quality assessment

The search and data extraction from the selected studies were carried out independently by two reviewers. When a discrepancy arose, it was resolved with the participation of a third reviewer. The essential information obtained included: title, authors, year of publication, study purpose, design type, language, sample size, age of participants, duration of the study, type of intervention, measurement tool, criteria, variable analyzed, results, statistical method, standard deviations, limitations, and conclusions.

Methodological quality assessment was performed independently by two evaluators using the PEDro scale (Physiotherapy Evidence Database) [11]. This tool allows assessing the risk of bias and methodological quality through a score ranging from 0 to 10. Scores of 9–10 represent excellent quality; 6–8, good; 4–5, moderate; and those below 4, poor quality [11].

The PEDro scale assesses internal validity (items 2–9) and the sufficiency of statistical information (items 10–11), excluding item 1 from the final calculation.

### Synthesis of results and meta-analysis

The integration of information was carried out through a narrative and descriptive synthesis. When feasible, data from the included studies were combined to conduct a meta-analysis. The primary variable analyzed in this analysis was the frequency of bowel movements, along with the evolution of other constipation symptoms.

Mean differences (MD) or standardized mean differences (SMD) with a 95% confidence interval were calculated. SMD were used due to the variability of the assessment tools used to measure the same clinical outcome. The effect size was classified according to Cohen's criteria: small (0.20), moderate (0.50), and large (0.80). Heterogeneity among studies was analyzed using the I^2^ statistic (*p* < 0.10). Values below 50% were low, 50% to 75% moderate, and above 75% high heterogeneity. A random-effects model was prioritized.

## Results

**Fig. 1:**
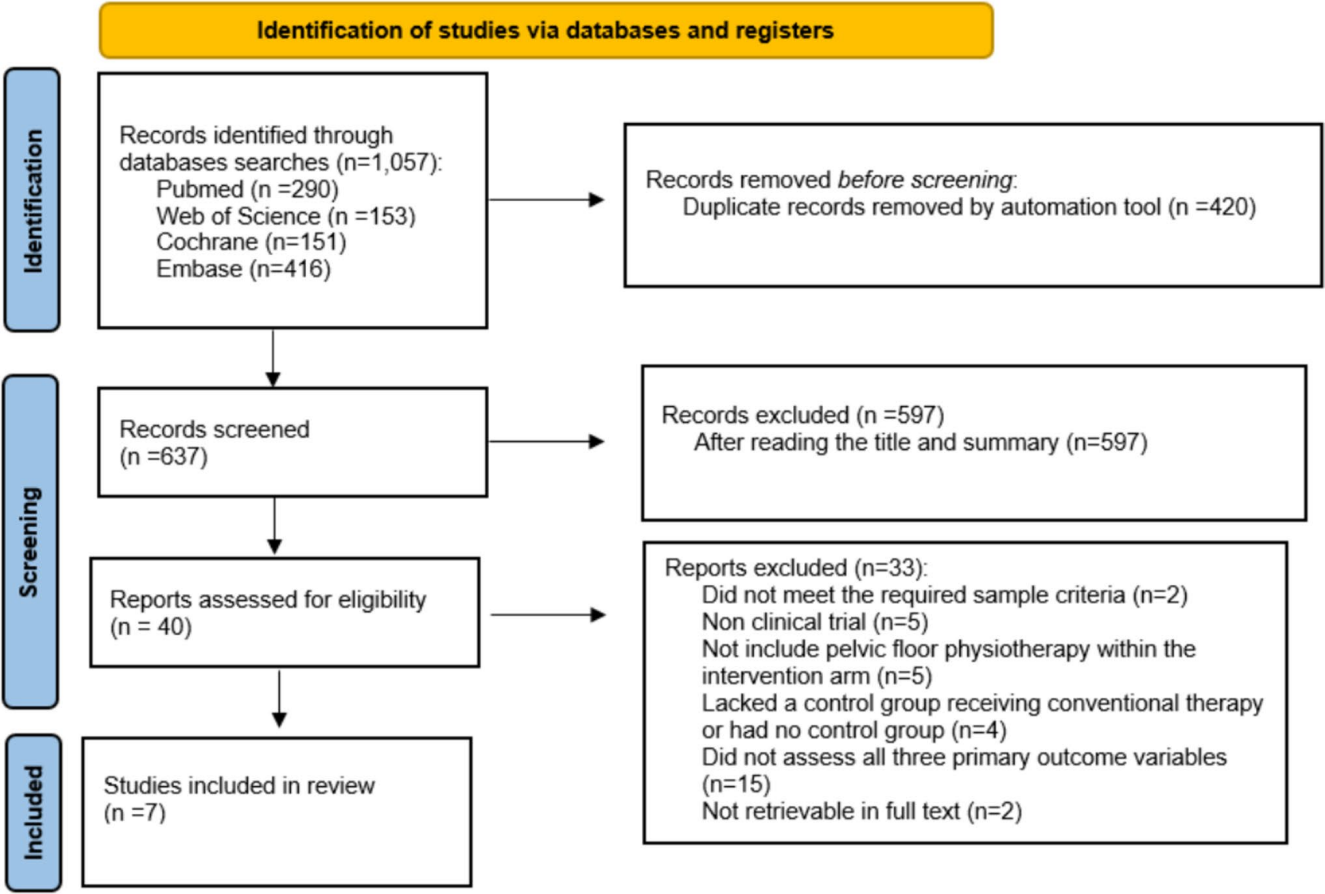
PRISMA flowchart

In the study selection process, 1,057 articles were initially identified. After removing 420 duplicates and 597 discarded titles/abstracts, 40 studies were selected for full-text review. Finally, 7 studies met the inclusion criteria and were incorporated into the systematic review (Fig. 1*. PRISMA Flowchart*).

### Methodological quality

The seven included clinical trials showed good methodological quality, with PEDro scores ranging between 6 and 8 (Annex 2. Methodological quality assessment. Available as Supplementary Material). The lowest scores were for the studies of Garag et al. [12], Ansari et al. [13], Soliman et al. [14] which obtained a score of 6; van Summeren et al. [15], Zakaryaei et al. [16], and Silva et al. [17] reached 7 points; and van Engelenburg et al. [18] was the highest rated, with 8 points.

The least compliant items were item 5 (blinding of participants) and item 6 (blinding of therapists), reflecting limitations related to controlling performance and detection bias.

### Qualitative synthesis of results

The interventions focused primarily on pelvic floor muscle exercises, sometimes combined with abdominal and respiratory training [12–16, 18]. Other approaches included electrotherapy [13], manual therapy [12–16, 18], stretching [13], and respiratory reeducation [14, 18]. The duration of interventions varied from 4 to 16 weeks, generally with 2 sessions per week [16, 17]. All control groups (GC) received conventional medical treatment based on osmotic laxatives and defecatory education [13–19]. (Annex 3. Characteristics of the included studies. Available as Supplementary Material).

#### Defecatory frequency

Five studies reported significant improvements in defecation frequency after physiotherapy. Garag et al. [12] observed favorable intergroup differences in the EG at the first and second month (*p* < 0.05). However, three studies [14, 15, 18] found no significant differences between groups. The evidence suggests a favorable trend of physiotherapy on defecatory frequency, although with inter-study heterogeneity.

#### Painful defecation

Physiotherapy showed a consistent beneficial effect on painful defecation. Significant reductions in pain were reported by Garag et al. [12] (*p* < 0.001) and Ansari et al. [13] (*p* = 0.037). van Engelenburg et al. [18] reported 100% therapeutic success in the EG vs. 58.8% in the control (*p* = 0.008).

#### Stool consistency

Results were variable based on the scale used (Bristol Stool Form Scale (BSFS) [19] or ROMA III). Garag et al. [12], van Engelenburg et al. [18], and Soliman et al. [14] reported improvements in consistency (*p* ≤ 0.001, *p* = 0.008, and *p* < 0.001, respectively). Zakaryaei et al. [16] described a greater proportion of normal consistency in the EG (88.5% vs 50%, *p* = 0.003) along with less PEG use (*p* < 0.0001). Overall, results suggest a moderate benefit of physiotherapy on this variable.

#### Quality of life

Most studies evidenced a significant improvement in quality of life in the EG, especially in the physical and emotional components. Garag et al. [12] observed significant improvements at three months (p ≤ 0.005). van Engelenburg et al. [18] detected improvement perceived by both children (*p* = 0.028) and parents (*p* = 0.047).

### Quantitative synthesis of results

**Fig. 2:**
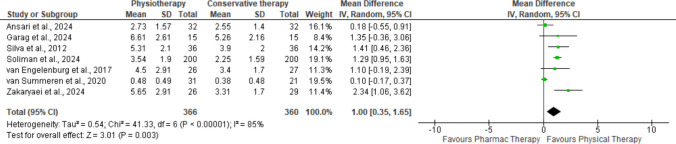
Random effects forest plot. Physiotherapy vs. conservative therapy

The analysis was carried out assuming a random effects model. The differences were statistically significant (MD = 1.00; 95% CI: 0.35 to 1.65; *p* = 0.003), indicating that, overall, physiotherapy is associated with better outcomes compared with conservative therapy. Heterogeneity is substantial (I^2^ = 85%), indicating considerable variability among studies that cannot be attributed to chance alone (Fig. 2) (Annex 4. Funnel Plot. Available as Supplementary Material). This variability implies differences in study populations, intervention protocols, outcome measurement, or methodological quality.

Overall, despite high heterogeneity, the aggregated evidence supports a beneficial effect of physiotherapy over conservative management.

## Discussion

### Global summary of findings

This systematic review included seven RCTs [12–18]. Overall, physiotherapy improves key symptoms such as pain during defecation, fecal consistency, and, to a lesser extent, quality of life. Results on defecatory frequency were less consistent.

In the meta-analysis comparing physiotherapy and conservative therapy for FC, the pooled SMD was 1 (95% CI: 0.35 to 1.65 − 1.53; *p* = 0.003), indicating a statistically significant overall effect in favour of physiotherapy. According to Cohen’s criteria, this value represents a large effect size, suggesting a clinically meaningful improvement. However, the heterogeneity across studies was high (I^2^ = 85%).

These overall findings should be interpreted with caution due to the differences among the included trials. Discrepancies could be related to pharmacological co-intervention, especially with the use of PEG. The available evidence grants a moderate recommendation for the use of physiotherapy as a complementary treatment within the multimodal approach to pediatric FC (Grading of Recommendations, Assessment, Development and Evaluation (GRADE) system). Its application appears especially beneficial in patients with dyssynergic defecation or insufficient response to conventional treatment.

### Interpretation by outcome variables

#### Defecatory frequency

Findings were heterogeneous. The ESPGHAN/NASPGHAN (European Society for Paediatric Gastroenterology, Hepatology and Nutrition/North American Society for Pediatric Gastroenterology, Hepatology and Nutrition) guidelines recommend PEG as the main treatment. The effect of physiotherapy is confirmed to be modest and dependent on the patient type [20, 21]. Physiotherapy’s utility might focus on optimizing defecatory function and improving adherence through telerehabilitation programs [14, 22, 23].

#### Painful defecation

The majority of trials [12–14, 16, 18] reported significant pain reductions. Pain relief is likely explained by improved pelvic floor coordination, reduced perineal hypertonia, and the learning of relaxation techniques, facilitating a more complete and less traumatic emptying [24]. Physiotherapy should be considered a key intervention for this symptom.

#### Stool consistency

Results were disparate. Normalization of consistency depends mainly on osmotic laxatives [24, 25], although physiotherapy can contribute to maintaining effects and reducing the necessary dose in the medium term. This suggests that physiotherapy acts as a therapeutic consolidation tool in the maintenance phase.

#### Quality of Life

The general trend points to a positive effect of physiotherapy. The majority of studies [12, 14, 18] showed significant improvements in quality of life. This reflects an integral benefit encompassing physical, emotional, and social aspects, reinforcing the value of physiotherapy in a biopsychosocial approach to pediatric FC.

#### Use of Instruments

The reviewed studies show wide variability in the instruments used. Some employed checklists [13] or Rome III criteria [14, 15, 18]. Validated scales used included the Visual Analogue Scale (VAS) [12, 26], BSFS [12, 16], Wong-Baker (16), Gastrointestinal Pediatric Quality of Life Inventory (GI PedsQL) [12, 27], Pediatric Quality of Life Inventory (PedsQL) [12], Defecation Disorder List [15], and Short Form-36 Health Survey (SF-36) [14]. This methodological heterogeneity evidences the need to unify criteria and validated instruments.

#### Plausible physiological mechanisms

The beneficial effects are supported by several physiological mechanisms:

1. Pelvic floor coordination retraining: favors muscle relaxation and corrects dyssynergic patterns [9, 28].
2. Abdominal muscle strengthening: increases intra-abdominal pressure and optimizes synergy [23].
3. Biofeedback: helps normalize rectal sensitivity and the anorectal inhibitory reflex [9].
4. Visceral mobilization: modulates colonic motility and decreases abdominal pain [29].

### Methodological strengths and limitations

#### Strengths

The exclusive inclusion of RCTs confers a high level of evidence. The methodological quality was assessed using the PEDro scale, with scores indicating moderate to high quality (6 to 8). Validated instruments such as the ROMA criteria, BSFS [19], PedsQL, GI PedsQL [27], and SF-36 were employed.

#### Limitations

Marked heterogeneity in the interventions (biofeedback, telerehabilitation, visceral mobilization, electrostimulation). Pharmacological co-intervention with PEG prevents isolation of physiotherapy's specific effect. Reduced sample sizes and follow-up shorter than 12 months restrict statistical power. The absence of blinding of patients and therapists represents a possible risk of bias.

### Clinical Implications and recommendations for future research

The findings support the use of physiotherapy as an adjuvant therapy in the treatment of pediatric FC, especially in patients with dyssynergia. Telerehabilitation is a viable and cost-effective alternative.

Future research should focus on multicenter RCTs with larger sample sizes, standardized intervention protocols, and homogeneous outcome measures. It is essential to evaluate long-term sustainability, cost-effectiveness, and the specific impact of each physiotherapeutic modality to determine the added value of physiotherapy.

## Conclusion

The results of this systematic review and meta-analysis suggest that pelvic floor physiotherapy can significantly improve symptom control in children and adolescents with FC, especially when combined with conventional medical treatment. Evidence indicates consistent effects on reducing pain during defecation and improving quality of life, while results on stool frequency and consistency are more variable and dependent on pharmacological co-intervention.

Conventional treatment with osmotic laxatives and defecatory education remains the basis of therapeutic management. However, physiotherapy emerges as an effective complementary tool to address the muscular and psychosocial components of the disorder.

The level of evidence is moderate (GRADE assessment), with a strong recommendation for its combined use with pharmacotherapy and health education.

Multicenter clinical trials with large samples, standardized protocols, and prolonged follow-up are required to confirm long-term effectiveness.

## Supplementary Information

Below is the link to the electronic supplementary material.

ESM 1 (DOCX 71.0 KB)

## Data Availability

All data supporting the findings of this study are available within the paper and its Supplementary Information. Data about mean, standard deviation an sample size, and the results of each included study in this review, are provided in Supplementary Table 3, along with original reference describing the results used in this study.
