# Supplementary material for: Efficacy of physiotherapeutic interventions in the management of functional constipation in pediatric and adolescent populations: a systematic review and meta-analysis
Source: Eur J Pediatr. 2026 Apr 10;185(5):251. doi: 10.1007/s00431-026-06831-8 (PMC13068696; doi:10.1007/s00431-026-06831-8)

**ANNEXES**

**Annex 1 – Table 1. Search strategies for the different databases**

The table details the search strings used in Pubmed, Cochrane, Embase, and Web of Science, combining terms such as "Functional Constipation," "Children," "Pelvic Floor Muscle Training," "Biofeedback," "Electrostimulation," and "Quality of Life" using Boolean operators “AND” and “OR”.

| Databases | Search strategies |
| --- | --- |
| **Pubmed** | - "Functional Constipation" AND Children AND (Biofeedback OR exercise OR physiotherapy OR "Quality of Life" OR "Interferential Electrical Stimulation" OR “conservative treatment”) - ("Constipation" OR "Functional Colonic Diseases" OR "Constipation" OR "Functional Constipation" OR "Chronic Constipation" OR "Pediatric Constipation" OR "Childhood Constipation") AND ("Physical Therapy Modalities" OR "Pelvic Floor Muscle Training" OR "Biofeedback, Psychology" OR "Exercise Therapy" OR "Pelvic Floor Rehabilitation" OR "Biofeedback Training" OR "Electrostimulation") AND ("Pelvic Floor" OR "Anal Canal" OR "Defecation" OR "Sphincter, Anal" OR "Pelvic Floor Dysfunction" OR "Anorectal Dysfunction" OR "Defecation Disorders" OR "Anal Sphincter") AND ("Pediatrics" OR "Children" OR "Infants" OR "Adolescents"). |
| **Cochrane** | - "Functional Constipation" AND Children AND (Biofeedback OR exercise OR physiotherapy OR "Quality of Life" OR "Interferential Electrical Stimulation" OR “conservative treatment”) - ("Constipation" OR "Functional Colonic Diseases" OR "Constipation" OR "Functional Constipation" OR "Chronic Constipation" OR "Pediatric Constipation" OR "Childhood Constipation") AND ("Physical Therapy Modalities" OR "Pelvic Floor Muscle Training" OR "Biofeedback, Psychology" OR "Exercise Therapy" OR "Pelvic Floor Rehabilitation" OR "Biofeedback Training" OR "Electrostimulation") AND ("Pelvic Floor" OR "Anal Canal" OR "Defecation" OR "Sphincter, Anal" OR "Pelvic Floor Dysfunction" OR "Anorectal Dysfunction" OR "Defecation Disorders" OR "Anal Sphincter") AND ("Pediatrics" OR "Children" OR "Infants" OR "Adolescents"). |
| **Embase** | - 'functional constipation' AND children AND ('biofeedback'/exp OR biofeedback OR 'exercise'/exp OR exercise OR 'physiotherapy'/exp OR physiotherapy OR 'quality of life'/exp OR 'quality of life' OR 'interferential electrical stimulation' OR 'conservative treatment'/exp OR 'conservative treatment') |
| **Web of Science** | - ("Constipation" OR "Functional Colonic Diseases" OR "Constipation" OR "Functional Constipation" OR "Chronic Constipation" OR "Pediatric Constipation" OR "Childhood Constipation") AND ("Physical Therapy Modalities" OR "Pelvic Floor Muscle Training" OR "Biofeedback, Psychology" OR "Exercise Therapy" OR "Pelvic Floor Rehabilitation" OR "Biofeedback Training" OR "Electrostimulation") AND ("Pelvic Floor" OR "Anal Canal" OR "Defecation" OR "Sphincter, Anal" OR "Pelvic Floor Dysfunction" OR "Anorectal Dysfunction" OR "Defecation Disorders" OR "Anal Sphincter") AND ("Pediatrics" OR "Children" OR "Infants" OR "Adolescents"). |

**Annex 2. Table 2. Methodological quality assessment.**

The table details the results of the assessment of the methodological quality of the incuded studies with the PEDro Scale.

|  | Eligibility criteria | Random allocation | Concealed allocation | Baseline comparability | Blind subjects | Blind therapists | Blind assessors | Adequate follow-up | Intention-to-treat analysis | Between-group comparisons | Point estimates and variability | Total score |
| --- | --- | --- | --- | --- | --- | --- | --- | --- | --- | --- | --- | --- |
| Garag et al.(12)  2024 | yes | yes | yes | yes | no | no | no | yes | no | yes | yes | 6/10 |
| Ansari et al.(13) 2024 | yes | yes | yes | yes | no | no | no | yes | no | yes | yes | 6/10 |
| van Summeren et al.(14)  2020 | yes | yes | yes | yes | no | no | yes | no | yes | yes | yes | 7/10 |
| van Engelenburg et al.(15)  2017 | yes | yes | yes | yes | no | no | yes | yes | yes | yes | yes | 8/10 |
| Soliman et al.(16)  2024 | yes | yes | no | yes | no | no | yes | yes | no | yes | yes | 6/10 |
| Zakaryaei et al.(17)  2024 | yes | yes | yes | yes | no | no | yes | yes | no | yes | yes | 7/10 |
| Silva et al.(18)  2013 | yes | yes | yes | yes | no | no | no | yes | yes | yes | yes | 7/10 |

**Annex 3 – Table 3. Characteristics of the studies included in the review.**

This table presents key data for the seven included RCTs, listing Study Type, Author, Year, PEDro score, Study Objective, Participants (N, Age), Intervention (EG and GC details), Measurements, and Results.

*(Specific data included for reference:* Garag et al.(12): N=45, PEDro 5/10, compared PEG, Physiotherapy, and Combined Treatment; van Engelenburg et al.(15): N=53, PEDro 8/10, compared Physiotherapy + PEG vs. Conventional Care + PEG; Soliman et al.(16): N=400, PEDro 7/10, compared Telerehabilitation + PEG vs. PEG; Arman et al.(17): N=55, PEDro 7/10, analyzed Visceral Manipulation + PEG vs. Conventional Care; etc.)*

| Author, Years, and PEDro | Study Objective | Participants | Intervention | Measurements Performed | Results |
| --- | --- | --- | --- | --- | --- |
| **Garag et al. (12) 2024**  ****5/10**** | **Analyze the short-term effects of traditional pharmacological treatment, physical therapy, and the combination of both in children with FE.** | N=45.  Age: 5-14.  EG1: N=15. EG2: N=15. CG: N=15. | **EG1 (Experimental group 1):** PEG (1.5 g/kg/day with a maintenance dose of 0.5 g/kg/day) and liquid paraffin (15-30 mL/day).  **EG2 (Experimental group 2):** Physical therapy: 20 minutes of interferential therapy + 5 to 10 minutes of connective tissue manipulation + breathing exercises + 8 to 10 minutes of lower limb and trunk stretching + 5 minutes of pelvic floor exercises.  **EG3 (Experimental group 3):** EG1+ EG2.  **Duration**: 3-month follow-up. | - **Defecation frequency.** - **Pain:** VAS. - **Gastrointestinal symptoms:** GI PedsQL. - **Stool consistency:** BSFS. - **Quality of Life:** PedsQL.   **Measurements:**   - T0: Baseline, - T1: 4 weeks, - T2: 8 weeks, - T3: 12 weeks. | **Post-Intervention Results (1, 2, and 3 months):**  **PedsQL:**   - **Post 1 month (between groups):** GC (70.94); GE1 (61.26).   P=0.009.   - **Post 2 months (between groups):** GE2 (79.24); GC (84.87); GA (61.42).   GE1 vs GE2: P=0.005; GE1 vs GC: P=0.001; GE2 vs GC: P=0.004.   - **Post 3 months (between grupos):** GE2 (80.22); GC (85.70); GE1 (60.33).   GE1 vs GE2: P=0.001; GE1 vs GC: P=0.001; GE2 vs GC: P=0.001.   - **P-value (Pre to Post 3 meses):** GE1 (P=0.081); GE2: P=0.001; GC: P=0.001.   **Defecation Frequency:**   - **Post 1 month (between groups):** GC (6.49); GE1 (4.76). P=0.038. - **Post 2 months (between groups):** GC (7.13); GE1 (4.94). P=0.026. - **Post 3 months (between groups):** No significant difference. - **P-value (Pre a Post):** GE1 (P=0.001); GE2 (P=0.001); GC (P=0.001).   **VAS:**   - **Post 5 days (between groups):** GE1 (5.83); GE2 (2.98); GC (2.96). P<0.001. - **Post 1 month (between groups):** GE1 (5.58); GE2 (2.31); GC (2.49). P<0.001. - **Post 2 months (between groups):** GE1 (5.40); GE2 (1.86); GC (2.33). P<0.001. - **Post 3 months (between groups):** GE1 (4.15); GE2 (1.38); GC (1.73). P<0.001. - **P-value (Pre a Post):** GE1 (P<0.001); GE2: P<0.001; GC: P<0.001.   **BSFS:**   - **Post 1 month (between groups):** GE1 (4.16); GC (3.95); GE2 (3.27).   GE1 vs GE2: P=0.001; GE1 vs GC: P=0.001; GE2 vs GC: P=0.009.   - **Post 2 months (between groups):** GE1 (4.22); GC (4.13); GE2 (3.21). P<0.001. - **Post 3 months (between grupos):** El GC (4.21); GE1 (3.66); GE2 (3.28).   GE1 vs GC: P=0.012; GE2 vs GC: P=0.008.   - **P-value (Pre a Post):** GE1: P<0.001; GE2: P=0.004; GC: P<0.001. |
| Ansari et al.(13)  2024  6/10 | **Evaluate the effectiveness of Kegel exercises compared to conventional therapy in the treatment of children with FE.** | N= 64.  Age: 8-18.  EG: N=32.  CG: N=32. | **CG:** Dietary training + defecation training + PEG (0.7 g/kg/day).  **EG:** CG + Kegel exercises: 10 fast contractions (2 sec contraction/1 sec relaxation) and 10 slow contractions (10 sec contraction/5 sec relaxation), 5 daily sets in the first week, increasing 5 sets each week. Performed in different positions. Sessions 50 to 60 minutes.  **Duration**: 3-month follow-up. | - **Defecation frequency.** - **Defecation time.** - **Laxative use.** - **Incomplete emptying.** - **Unsuccessful defecation.** - **Abdominal pain.** - **Painful defecation.**   All evaluated using a self-care checklist.  **Measurements:**   - T0: Baseline, - T1: 12 weeks. | 1. **Defecation time.**  - Pre (between groups): P=0.321. - Post (between groups): P<0.001. - P-value (pre y post): GE <0.001 y GC 0.002.  1. **Incomplete emptying**.  - Pre (between groups): P=0.112. - Post (between groups): P=0.001. - P-value (pre y post): GE <0.001 y GC:0.001.  1. **Unsuccessful defecation.**  - Pre (between groups): P=0.131. - Post (between groups): P=0.001. - P-value (pre y post): GE <0.001 y GC 0.005.  1. **Abdominal pain.**  - Pre (between groups): P=0.512. - Post (between groups): P=0.001. - P-value (pre y post): GE y GC <0.001.  1. **Painful defecation**.  - Pre (between groups): P=0.083. - Post (between groups): P=0.037. - P-value (pre y post): GE y GC <0.001.  1. **Defecation frequency**:  - Pre (between groups): P=0.103. - Post (between groups): P=0.866. - P-value (pre y post): GE y GC <0.001.  1. **Laxative use**:  - Pre (between groups): P= 0.478. - Post (between groups): P=0.659. - P-value (pre y post): GE 0.138 y GC: 0.522. |
| van Summeren et at.(14)  2020  7/10 | **Determine the efficacy of Physical Therapy compared to conventional treatment in children and adolescents with FE.** | N=134.  Age: 4-17.    EG: N=67.  CG: N=67. | **CG:** Sphincter control + nutritional counseling + laxatives.  **EG:** CG + Pelvic Floor/Pediatric Physical Therapy: training + defecatory re-education. 9 sessions of 30 minutes.  **Duration:** 4th and 8th month follow-up. | **Rome III criteria (without laxatives for 4 weeks) – considered positive if ≥1 is not met:**  - < 3 bowel movements per week.  - Hard stools ≥25%.  - Incomplete evacuation ≥25%.  - Straining during defecation ≥25%.  - Sensation of obstruction ≥25%.  - Manual maneuvers ≥25%.  **Quality of Life:**  - Defecation checklist (emotional and social subdomains).  **Perceived Global Effect:**  - Structured interview.  **Measurements**:   - T0: baseline. - T1: week 16. - T2: week 32. | **Absence of FI without laxatives:**   - Adjusted Relative Risk (aRR): 0.80 (95% CI: 0.44–1.30); p = 0.397. - Success at 4 months: EG 17% vs. CG 28%. - Success at 8 months: EG 42% vs. CG 41%.   **Absence of FI (with or without laxatives):**   - aRR: 1.12 (95% CI: 0.82–1.34); *p* = 0.405. - Success at 4 months: EG 68% vs. CG 64%. - Success at 8 months: EG 73% vs. CG 61%.   **Quality of life (median, Interquartile Range):**   - 4 months: EG 82 (75–88), CG 84 (74–88). - 8 months: EG 85 (79–92), CG 85 (77–90). - p = 0.675.   **Perceived global effect:**   - aRR: 1.40 (95% CI: 1.00–1.73); p = 0.048. - Improvement at 4 months: EG 63% vs. CG 38%. - Improvement at 8 months: EG 62% vs. CG 52%.   **Per-protocol analysis – Success (absence of FI):**   - Without laxatives: aRR 0.88 (95% CI: 0.60–1.13). - With or without laxatives: aRR 0.98 (95% CI: 0.53–1.56).   **Prognosis of success at 8 months – associated factors (Models 1 and 2):**   - **Abdominal pain ≥1/week:**M1: OR 0.5 (0.2–1.1); p = 0.083. M2: OR 0.3 (0.1–0.8); p = 0.017. - **Stool retention:** M1: OR 0.3 (0.1–0.8); p = 0.012. M2: OR 0.2 (0.1–0.4); p < 0.001. - **FI ≥1/week:** M1: OR 0.5 (0.2–1.1); p = 0.082. M2: OR 0.1 (0.1–0.5); p < 0.001. - **Hard stools or painful defecation:** M1: OR 0.9 (0.4–2.2); p = 0.872. M2: OR 0.5 (0.2–1.3); p = 0.141. - **Defecation frequency:** M1: OR 0.8 (0.3–2.5); p = 0.718. M2: OR 1.1 (0.3–3.4); p = 0.895. - **Large stools obstructing the toilet:** M1: OR 0.7 (95% CI: 0.2–2.2); p = 0.565. M2: OR 0.9 (95% CI: 0.3–2.6); p = 0.801. |
| van Engelenburg et al.(15)  2017  8/10 | **Analyze the efficacy of Physical Therapy in contrast to usual medical care in children and adolescents with FE.** | N=53.  Age: 5-16.  EG: N=26.  CG: N=27. | **CG:** Education + sphincter control + dietary counseling + PEG.  **EG:** CG + Physical Therapy: specific abdominal and pelvic floor training + postural and defecatory re-education + respiratory re-education + body awareness work. PEG adjusted when necessary.  **Duration:** 6-month follow-up. | **Absence of FI (Rome III criteria):**   - < 3 bowel movements per week. - Hard stools ≥25%. - Incomplete evacuation ≥25%. - Straining during defecation ≥25%. - Sensation of obstruction ≥25%. - Manual maneuvers ≥25%.   **Perceived global effect**  **Pelvic floor dysfunction and motor control:**   - Assessment of muscle dysfunction and motor control   **Quality of life:**   - Numeric Rating Scale (NRS) - Strengths and Difficulties Questionnaire (SDQ)   **Measurements:**  Baseline and end of study. | **EG vs. CG:**   - **Treatment success (absence of FI):** 92.3% EG vs. 63.0% CG; p = 0.011, - **Absence of all Rome III criteria post-intervention:** 92.3% EG vs. 44.4% CG; p = 0.001. - **Discontinuation of PEG use:** 65.4% EG vs. 37.0% CG; p = 0.009. - **Perceived global effect (parents):**88.5% EG vs. 33.3% CG; p < 0.001. - **Parents NRS (absolute change):** +1.8 points EG (standard deviation **(**SD): 3.1) vs. CG (SD: 2.9); p = 0.047. - **Children NRS (absolute change):** +2.0 points EG (SD: 3.2) vs. CG (SD: 2.9); p = 0.028. - **SDQ:** No significant differences; p = 0.78 (EG SD: 0.3; CG SD: 3.3).   **Individual Rome III criteria post-intervention - significant improvements in EG:**   - **Hard stools/painful defecation:** 15/15 EG vs. 10/17 CG; p = 0.008. - **Stools blocking the toilet:** 15/15 EG vs. 8/12 CG; p = 0.042.   **Rome III criteria without significant differences between groups:**   - < 3 bowel movements per week: p = 0.25. - FI: p = 0.39. - Stool withholding: p = 0.39. - Fecal impaction: p = 0.19. |
| Soliman et al.(16)  (2024)  7/10 | **Assess the effectiveness of a home telerehabilitation program versus usual pharmacological treatment + medical conservative treatment in children and adolescents with FE.** | N=400.  Age: 4-18.  EG: N=200. CG: N=200. | **CG:** Diet regimen and PEG (0.7 g/kg per day).  **EG:** CG + Telerehabilitation program: isometric abdominal muscle training + breathing exercises + abdominal massage.  **Duration:** 6-month follow-up. | **Absence of Pelvic Floor Dyssynergia (Rome III Criteria):**  Diagnosis made using the Rome III questionnaire  Criteria:   - < 3 bowel movements per weeK. - Hard stools ≥ 25% of the time. - Sensation of incomplete evacuation ≥ 25%. - Straining during defecation ≥ 25%. - Sensation of anorectal obstruction/blockage ≥ 25%. - Need for manual maneuvers to facilitate defecation ≥ 25%.   **Quality of Life:**   - SF-36 questionnaire.   **Measurements:**   - At the beginning and end of the study.   . | **Significant Improvements in the EG vs. CG (Rome III Criteria):**  **Symptoms (absence according to the Rome III Questionnaire):**   - **Defecation frequency (< 2/week):** Between groups: p < 0.001. Within groups: EG < 0.001; CG = 0.001. - **Hard stools:** p < 0.001 in all analyses - **Duration of hard stools:** Between groups: p = 0.025. Within groups: EG < 0.001; CG = 0.009. - **Pain during defecation:** Between groups: p < 0.001. Within groups: EG < 0.001; CG = 1.0 (ns). - **Urgency (running to the bathroom):** Between groups: p = 0.314 (ns). Within groups: EG < 0.001; CG < 0.001. - **Straining during defecation:** p < 0.001. - **Mucus/phlegm in stool:** p < 0.001. - **Incomplete evacuation:** p < 0.001. - **Stools that clog the toilet:** Between groups: p = 0.001. Within groups: EG < 0.001; CG < 0.001. - **Voluntary stool retention (at home):** Between groups: p = 0.003. Within groups: EG < 0.001; CG = 0.33 (ns). - **Fecal impaction:** Between groups: p = 0.001. Within groups: EG < 0.001; CG = 0.21 (ns). - **Staining of underwear (frequency, amount, duration):** p < 0.001 in all analyses.   **Quality of Life Improvements (SF-36):**  **Domains with significant differences (EG > CG; between-group p < 0.001):** Physical functioning, physical role, emotional role, bodily pain, general health, vitality, and social functioning.  **Domains without significant differences between groups:**   - **General health:** improved in both groups, but no between-group difference (as noted in discussion). - **Mental health:** no significant difference between EG and CG.   - Within groups: EG < 0.001; CG < 0.001.   - Post-intervention SD: EG = 5.16; CG = 4.52.   **Bodily pain (post-intervention standard deviation):**   - EG = 13.15. - CG = 16.17. |
| Arman et al.(17)  2024  7/10 | **Analyze the impact of visceral manipulation in children and adolescents with FE who do not show improvement with medical conservative treatment…** | N=55  Age: 5- 18.  EG: N=26.  CG: N=29. | **GC:** Education + diet rich in fiber and liquids + PEG (0.3-0.8 g/kg/day).  **EG:** GC + Visceral and abdominal mobilization. 2 sessions of 40 minutes/week. **Duration:** 4-week follow-up. | - **Abdominal and defecation pain:** Wong-Baker Scale. - **Stool consistency:** BSFS. - **Defecation frequency:** Number of bowel movements/week. - **Laxative use:** PEG dose.   **Measurements:** beginning and 4 weeks. | **Improvements in the EG vs. CC:**  - Pain during defecation (Wong–Baker FPRS).   - Post-treatment (between groups): p = 0.002. - Pre–post comparison: EG p < 0.001; CG p < 0.001. - Post-treatment SD: EG = 1.87; CG = 2.48.   - Abdominal pain (Wong–Baker FPRS).   - Post-treatment (between groups): p = 0.002. - Pre–post comparison: EG p < 0.001; CG p = 0.026. - Post-treatment SD: EG = 1.95; CG = 2.40.   - BSFS.   - Post-treatment (between groups): p = 0.32 (not significant). - Pre–post comparison: EG p = 0.01; CG p = 0.002. - Post-treatment SD: EG = 0.85; CG = 1.70. - % with normal stool consistency (types 3–5): EG 88.5% vs. CG 50% (p = 0.003).   -Defecation frequency (n/week).   - Post-treatment (between groups): p = 0.001. - Pre–post comparison: EG p < 0.001; CG p < 0.001. - Post-treatment SD: EG = 2.91; CG = 1.70.   - PEG dose (g).   - Post-treatment (between groups): p < 0.0001. - Pre–post comparison: EG p = 0.002; CG p = 0.77 (not significant). - Post-treatment SD: EG = 2.36; CG = 11.83. - Significantly lower post-treatment PEG dose in the EG. |
| Silva et al.(18)  2013  7/10 | **Compare the effect of muscle training + abdominal massage + diaphragmatic breathing with conventional medical treatment in children and adolescents with FE.** | N= 72.  Age: 4-18.  EG: N=36.  CG: N=36. | **CG:** Magnesium hydroxide (minimum 2 ml/kg) + sphincter control + fiber-rich diet and liquid..  **EG:** CG + isometric abdominal training (sets of 3 contractions/relaxations of 10 seconds, increasing to 5 reps between 3rd and 6th week) + diaphragmatic breathing exercises (2 sets of 10 repetitions) + abdominal massage. 2 sessions of 40 minutes/week.  **Duration:** 6-week follow-up. | - **Defecation frequency.** - **Retentive IF.** - **Straining and pain during defecation.** - **Retention behavior.** - **Stool consistency.**   All measured with a bowel habit diary.  **Measurements**: T0 to T6 (weekly). | **Post-Treatment Results (After 6 Weeks)**   1. **Defecation frequency (days/week):**    - Mean ± SD: EG: 5.1 ± 2.1; CG: 3.9 ± 2.0.    - Between-group comparison (post): p = 0.01. 2. **FI (days/week):**    - Mean ± SD: EG: 3.6 ± 1.9; CG: 3.0 ± 2.1.    - Between-group comparison (post): p = 0.31. 3. **Straining during defecation:**    - Yes: EG: 17 (47.2%); CG: 16 (44.4%).    - No: EG: 19 (52.8%); CG: 20 (55.6%).    - Between-group comparison (post): p = 1.00. 4. **Painful defecation:**    - Yes: EG: 9 (25.0%); CG: 10 (27.7%).    - No: EG: 27 (75.0%); CG: 26 (72.3%).    - Between-group comparison (post): p = 1.00. 5. **Stool consistency:**    - Hard/dry: EG: 15 (41.7%); CG: 19 (52.8%).    - Soft/liquid: EG: 21 (58.3%); CG: 17 (47.2%).    - Between-group comparison (post): p = 0.48. 6. **Retention behavior:**    - Yes: EG: 4 (11.1%); CG: 8 (22.2%).    - No: EG: 32 (88.9%); CG: 28 (77.8%).    - Between-group comparison (post): p = 0.34. |

**Annex 4 – Funnel Plot**


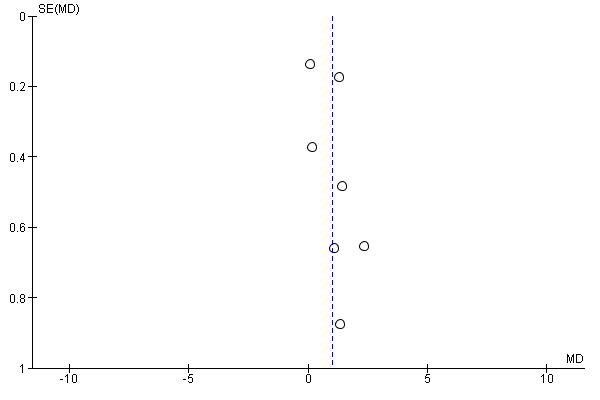

Supplement: Supplementary file 1 — (DOCX 71.0 KB) [file 431_2026_6831_MOESM1_ESM.docx]
